# Vpx complementation of ‘non-macrophage tropic’ R5 viruses reveals robust entry of infectious HIV-1 cores into macrophages

**DOI:** 10.1186/1742-4690-11-25

**Published:** 2014-03-21

**Authors:** Petra Mlcochova, Sarah A Watters, Greg J Towers, Mahdad Noursadeghi, Ravindra K Gupta

**Affiliations:** 1 Department of Infection, University College London, London, UK; 2 MRC/UCL Centre for Medical Molecular Virology, 90 Gower St, WC1E 6BT London, UK

**Keywords:** Transmitted/founder viruses, HIV, Macrophages, Vpx, Entry, Reverse transcription

## Abstract

**Background:**

It is now known that clinically derived viruses are most commonly R5 tropic with very low infectivity in macrophages. As these viruses utilize CD4 inefficiently, defective entry has been assumed to be the dominant restriction. The implication is that macrophages are not an important reservoir for the majority of circulating viruses.

**Results:**

Macrophage infection by clinical transmitted/founder isolates was 10-100 and 30-450 fold less efficient as compared to YU-2 and BaL respectively. Vpx complementation augmented macrophage infection by non-macrophage tropic viruses to the level of infectivity observed for YU-2 in the absence of Vpx. Augmentation was evident even when Vpx was provided 24 hours post-infection. The entry defect was measured as 2.5-5 fold, with a further 3.5-10 fold block at strong stop and subsequent stages of reverse transcription as compared to YU-2. The overall block to infection was critically dependent on the mechanism of entry as demonstrated by rescue of infection after pseudotyping with VSV-G envelope. Reverse transcription in macrophages could not be enhanced using a panel of cytokines or lipopolysaccharide (LPS).

**Conclusions:**

Although the predominant block to clinical transmitted/founder viruses is post-entry, infectivity is determined by Env-CD4 interactions and can be rescued with VSV-G pseudotyping. This suggests a functional link between the optimal entry pathway taken by macrophage tropic viruses and downstream events required for reverse transcription. Consistent with a predominantly post-entry block, replication of R5 using viruses can be greatly enhanced by Vpx. We conclude therefore that entry is not the limiting step and that macrophages represent clinically relevant reservoirs for ‘non-macrophage tropic’ viruses.

## Background

Macrophages are thought to be important cellular targets of HIV infection due to their access to multiple tissue compartments and relative longevity even when productively infected with HIV (reviewed in [1]). There has been renewed interest in HIV infection of macrophages across clinical and basic science disciplines, driven by two factors. Firstly, recognition of neurocognitive disease in the post HAART era [2] and its association with compartmentalized CNS viral replication [3-5]. Secondly, the rise of the ‘eradication’ agenda will inevitably need to address macrophages as intermediate or long-term reservoirs [5,6].

Early observations that some CCR5 using laboratory viruses such as YU-2 (cloned directly from the central nervous system tissue) efficiently infected macrophages led to conflation of the terms CCR5 tropism and macrophage tropism [7]. This dogma has been challenged by work demonstrating that R5 tropic envelopes are diverse in their ability to replicate in monocyte derived macrophages [8]. More specifically, envelope glycoproteins (Envs) from viruses isolated from central nervous system often confer efficient macrophage replication upon pseudotyping viruses as compared to Envs from blood or lymph nodes in patients with neurocognitive disease [9,10], and since then a diverse range of envelope determinants have been implicated in this phenotype, often associated with the CD4 binding site [11-13].

As the accuracy of cloning of HIV-1 *env* may be compromised by both recombination and biased sampling [14], more recent studies have used single genome amplification (SGA) to overcome this limitation [15,16]. Furthermore, use of Env alone neglects the role of other parts of the genome in envelope expression and infectivity, for example Nef and Gag [17-19]. Recently, and after significant investment, a panel of clinical full genome subtype B and C isolates has become available [15,20-22]. The sequences are from early infection and have been derived using SGA and sequencing. These clones represent the most appropriate clinical isolates for HIV research gained thus far. Studies on these viruses have shown that inefficient macrophage infection (compared to prototypic macrophage tropic viruses) is prevalent, and indeed the norm in both transmitted founder (T/F) viruses and viruses derived during chronic infection [15,21,23]. Given that viral determinants map to Env, the assumption has been that the entry event represents the dominant restriction.

SAMHD1 was recently identified as an anti-HIV restriction factor in myeloid lineage cells (dendritic cells and macrophages) acting at post-entry step of the virus life-cycle [24,25]. SAMHD1 is a dNTP hydrolase thought to limit reverse transcription (RT) through decreasing levels of dNTPs [26]. This mammalian protein is the target of the lentiviral accessory gene *vpx*, found in HIV-2/SIVsm lineage viruses, but not in the SIVgsn lineage or HIV-1/SIVcpz [24]. Vpx, akin to other viral countermeasure proteins such as Vpu and Vif, degrades its target restriction factor via a ubiquitin dependent proteasomal pathway [24,25].

In this study we demonstrate that infectivity of a panel of ‘non-macrophage tropic’ clinical isolates is rescued by Vpx complementation in macrophages. We show that the dominant restriction is at early RT and that this infection block is determined by Env-CD4 interactions and can be rescued with VSV-G pseudotyping.

## Results

### Clinically derived transmitted/founder viruses are rescued by Vpx

**Figure 1:**
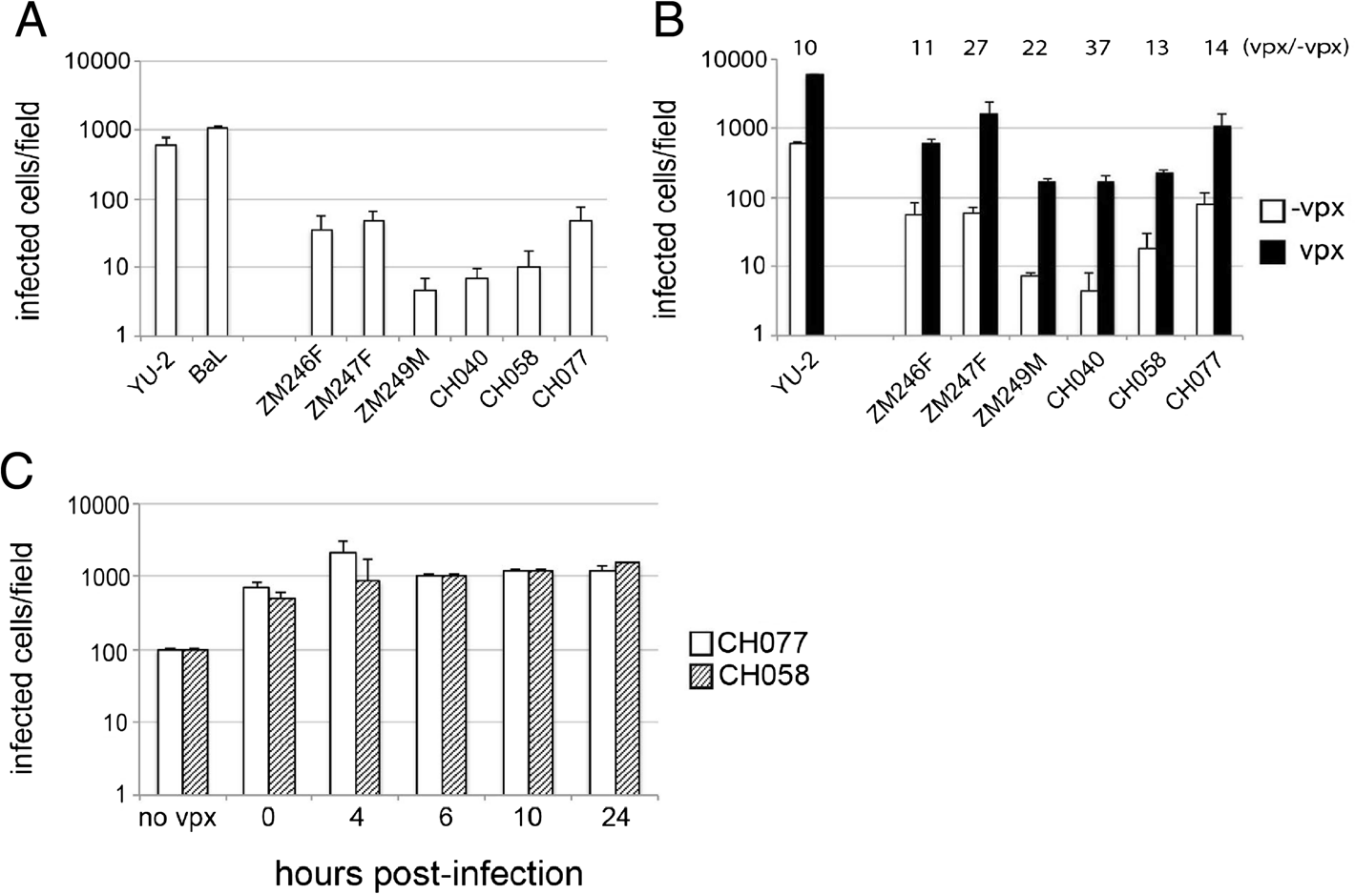
**Vpx rescues T/F viruses to a similar extent as that seen for YU-2 in macrophages. (A)** Monocyte derived macrophages (MDM) were infected with equal amounts of p24 of macrophage tropic viruses YU-2, BaL and subtype C and B full-length viruses for 6 h. Cells were washed and new medium was added. MDM were fixed and labeled for intracellular p24 48 h post-infection. Data shown are mean of three independent experiments and error bars represent the standard deviation. **(B)** MDM were infected as in **(A)** with or without 1 ng of SIVmac particles containing Vpx. Cells were washed after 6 h and new medium was added. Intracellular p24 staining was analyzed 48 h post-infection. Graph shows representative data of two independent experiments each in duplicates. Error bars represent the standard deviation. White bars: infection without Vpx; black bars: infection with Vpx. Numbers above the graph show fold difference between infection in presence (vpx) and absence (-vpx) of vpx VLP. **(C)** MDM were infected with CH077 (white bars) and CH058 (checked bars) clinically derived viruses for 4 h and Vpx was added at different time points. All MDM were fixed and stained for intracellular p24 at 48 h post-infection. No Vpx: MDM were infected for 4 h, washed and new medium added. 0 h: MDM were infected with virus and complemented with Vpx at the same time for 4 h, washed and new medium was added; 4 h, 6 h, 10 h, 24 h: MDM were infected with virus for 4 h, washed and new medium was added; Vpx was added at 4 h, 6 h, 10 h and 24 h following the infection. Graph shows representative data of two independent experiments each in duplicates. Error bars represent the standard deviation.

The available panel of three subtype C and three subtype B full-length clinically derived viruses (Table 1) were derived from plasma in individuals with acute infection [27] following mucosal transmission, representing transmitted/founder (T/F) viruses [15]. These viruses were previously reported to replicate poorly in spreading infection in MDM [15,23]. We show here that in contrast to T cells (where similar infectivity was detected across all viruses tested; Additional file 1: Figure S1), all clinically derived viruses tested in MDM showed lower infection when compared to macrophage tropic viruses such as YU-2 or BaL over a single round of infection. Using intracellular p24 staining 48 h post-infection, we detected 10-100 or 30-450 fold lower infection of MDM for T/F viruses compared to YU-2 or BaL, respectively (Figure 1A). Furthermore, we found substantial differences between clinical isolates, for example ZM249M and CH040 differed by almost one order of magnitude compared to ZM246F, ZM247F or CH077. Nevertheless, there was no statistically significant difference in MDM infection between subtype B and C clinically derived viruses (p = 0.70).

**Table 1:** Details of single genome derived infectious molecular clones and host demographic characteristics

**Virus**	**Location of patient**	**Gender**	**Transmission type**	**Fiebig stage**	**HIV-1 subtype**
ZM246F	Zambia	F	Heterosexual	II	C
ZM247F	Zambia	F	Heterosexual	II	C
ZM249M	Zambia	M	Heterosexual	IV	C
CH040	USA	M	MSM	II	B
CH058	USA	M	MSM	II	B
CH077	USA	M	MSM	II	B

Key: MSM: men who have sex with men; Fiebig stage is a serological staging system for defining the timing of infection.

We tested the sensitivity of T/F viruses to Vpx (Figure 1B) by adding VSV-G pseudotyped SIV virions containing Vpx to MDM at the same time as virus inoculum. Six hours post-infection cells were washed and new medium was added to the culture. Complementation with Vpx increased infection of MDM by at least one order of magnitude for all viruses tested, including YU-2. Interestingly, Vpx complementation augmented macrophage infection for three of the T/F viruses (ZM246F, ZM247F and CH077) to the level of YU-2 in the absence of Vpx (Figure 1B). We further explored whether infection of MDM could be rescued after virus inoculation. We infected MDM with two clinically derived viruses (CH058 and CH077) and complemented with Vpx at various intervals post-infection (4, 6, 10, 24 h). Cells were fixed and stained for intracellular p24 protein 48 h post-infection. We observed a rescue mediated by Vpx at all time points tested up to 24 h post-infection (Figure 1C) in these experiments. These data demonstrate that (i) clinically derived T/F viruses are as sensitive to Vpx as the widely used macrophage tropic strain (YU-2), (ii) the rescue of T/F viruses infection mediated by Vpx can be very potent and can reach infection similar to macrophage tropic viruses, (iii) rescue can be achieved even 24 hours post-infection. Nonetheless, Vpx augmentation did not abrogate the single round replication difference between YU-2 and clinical strains, consistent with an independent block.

### Macrophage entry is only modestly impaired in ‘non-macrophage tropic’ isolates

It was suggested, though not proven, that the T/F virus phenotype of low replication capacity in macrophages maps probably to a virus entry defect. That notion is paradoxical to our data showing potent Vpx rescue infection. We decided to (i) investigate if T/F virus infection is dependent on cell surface CD4 levels (as previous work suggested that the T/F strains are less sensitive to soluble CD4 [23]) and (ii) directly measure entry efficiency of full-length T/F viruses in both T cell lines and MDM.

**Figure 2:**
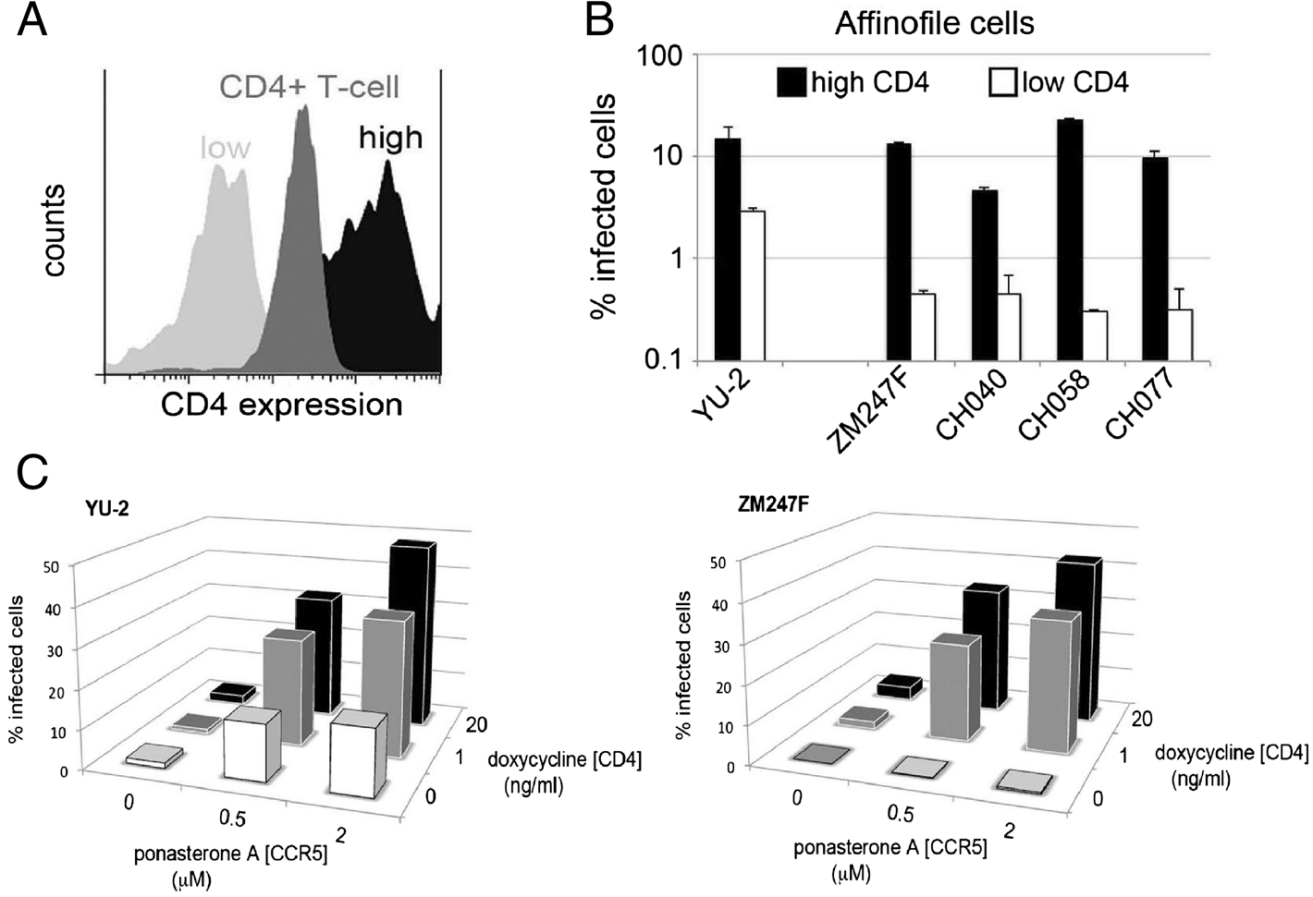
**Full-length transmitted/founder viruses use low levels of the cells surface CD4 inefficiently. (A)** A representative flow cytometry experiment showing CD4 expression levels on minimally (**low**) and maximally (**high**) induced 293-Affinofile cells in comparison with CD4+ primary T cells. **(B)** Equal amounts of p24 of YU-2 and clinically derived transmitted viruses were used to infect 293-Affinofile cells, which were maximally induced to express high levels of CCR5 and high (**black bars**) or low (**white bars**) levels of CD4. Cells were washed, fixed, permeabilized with saponin and stained with anti-HIV-1 p24 FITC-conjugated monoclonal antibody 48 h post-infection. Percentage infection was determined by flow cytometry. Data are representative of at least two independent experiments and error bars represent the standard deviation. **(C)** 293-Affinofile cells were induced to express different cell surface levels of CD4 and CCR5. Cells were infected with 50 ng of p24 of YU-2 and ZM247F and percentage of infection determined by flow cytometry. Data shown are representative example of three independent experiments.

Macrophages are known to express lower levels of CD4 than CD4+ T cells [28], and envelopes from ‘non-macrophage tropic’ HIV strains have been reported to utilize these low levels inefficiently (reviewed in [29]). We tested if the replication block detected in MDM is dependent on CD4 cell surface levels. We used 293-Affinofile cells whose surface levels of CD4 and CCR5 can be manipulated pharmacologically. These cells were maximally induced to express CCR5 at the cell surface and at the same time induced to express low or high levels of CD4 (Figure 2A,B), as previously described [30]. Cells were infected with normalized virus stocks and infection measured 48 h later by flow cytometry. All clinically derived viruses as well as YU-2 were able to infect cells with high levels of CD4 to the same extent (Figure 2B, black bars). Nevertheless, in target cells expressing low levels of CD4 (Figure 2B, white bars), infection was reduced by approximately ten fold in clinical isolates as compared to YU-2, largely recapitulating findings in macrophages (Figure 1A). To demonstrate dependence of virus infection on both CD4 and CCR5 levels in this assay system, we used YU-2 and ZM247F viruses to infect 293-Affinofile cells (Figure 2C) expressing variable levels of both receptors. Infection by both viruses was sensitive to increases in both CD4 and CCR5 expression levels. However, at the lowest level of CD4 expression, increasing CCR5 levels could partially rescue infection by YU-2 (10 fold increase) but not ZM247F (Figure 2C). These results suggest that the overall block to infection in macrophages is sensitive to CD4 cell surface levels.

**Figure 3:**
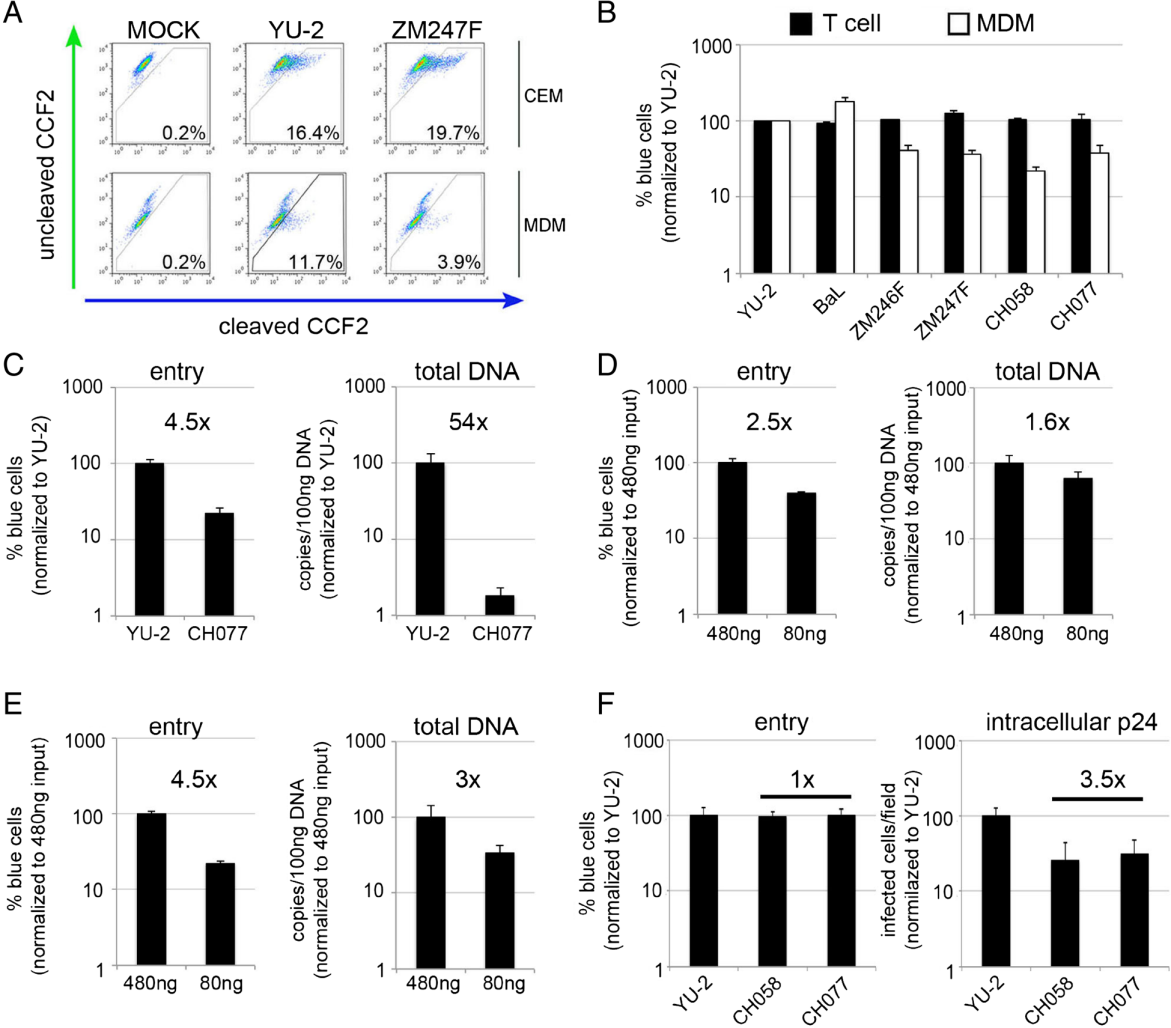
**Transmitted/founder viruses display modestly reduced entry efficiency in MDM. (A,B)** CEM.NKR-CCR5-Luc cells (CEM) or MDM were infected with equal amounts of p24 of BlaM-Vpr containing viruses for 4 h. Cells were loaded with CCF2/AM dye and fusion events were detected by flow cytometry using BD LSR Fortessa, and gated from 10,000 cells. **(A)** A representative example of three independent experiments. Percentage in each panel represents virus fusion positive cells (cleaved CCF2). **(B)** Graph shows a percentage of detected fusion events (blue cells) normalized to YU-2 as a control (100%), and represents an average of three independent experiments, each conducted in triplicate. Black bars represent CEM cells and white bars MDM. **(C)** MDM were infected with equal amounts of p24 of BlaM-Vpr containing viruses for 4 h. Cells were inspected for virus entry as described above, at the same time cells were harvested at 6 h post-infection for total DNA isolation and total viral DNA was determined using quantitative PCR.% blue cells or copies/100 ng DNA are normalized to YU-2 (100%). **(D,E)** MDM were infected with equal amounts of p24 of YU-2 **(D)** or BaL **(E)** BlaM-Vpr containing viruses for 4 h. Cells were inspected for virus entry as described above, at the same time cells were harvested at 6 h post-infection for total DNA isolation and total viral DNA was determined using quantitative PCR.% blue cells or copies/100 ng DNA are normalized to higher p24 input of virus (480 ng ~ 100%). **(F)** MDM were infected with different amounts of p24 of BlaM-Vpr containing viruses to achieve equal virus entry for 4 h. Inspected for entry efficiency and also labeled for intracellular p24 48 h post-infection.% blue cells and infected cells/field are normalized to YU-2 (100%). **(C-F)** Data shown are representative example of at least two independent experiments conducted in duplicates. Error bars represent the standard deviation.

To investigate the entry efficiency of full-length T/F viruses in both the T cell line CEM.NKR-CCR5-Luc and MDM we used the well established BlaM-Vpr assay [31]. We consistently found a 2.5-4.5 fold reduction in macrophage entry but not T cell entry when comparing T/F viruses to YU-2 and BaL (Figure 3A and B). As there was at least 10-100 fold (compared to YU-2) or 30-450 fold (compared to BaL) difference in single round macrophage infection (Figure 1A,B), this modest entry defect implied a possible further post-entry block to the T/F viruses. Also, measurement of total viral DNA using qPCR showed a much larger difference between YU-2 and a representative T/F virus CH077 (54 fold difference) when entry was again only modestly affected (4.5 fold, Figure 3C).

To rule out sensitivity of the entry assay as a contributory factor in the observed phenotype, we firstly aimed to demonstrate a direct and consistent correlation between fusion and accumulation of RT products for a given virus across a range of input doses and thereby a range of entry event frequencies. In this experiment BlaM-Vpr containing viruses were used to infect MDM at two different input doses (80 and 480 ng of p24 as determined by p24 ELISA). We detected a 2.5 fold difference in virus entry and 1.6 fold difference in total viral DNA between the two doses of YU-2 BlaM-Vpr virus used (p = NS) (Figure 3D). Similarly, BaL BlaM-Vpr virus showed a 4.5 fold difference in virus entry assay and a 3 fold difference in total DNA between the two viral doses (p = NS) (Figure 3E). Secondly, we excluded non-envelope mediated entry into MDM using Env-deficient HIV-1 subtype C ZM247Fv1Δenv virus and ZM247Fv1Δenv complemented *in trans* with YU-2 envelope (Additional file 2: Figure S2). Finally, we adjusted p24 amounts of YU-2 and two T/F viruses (CH077 and CH058) to achieve the same entry efficiency and measured intracellular p24 in infected MDM 48 h later. Interestingly, even though entry was the same we still detected an additional block to infection (3-4 fold) between YU-2 and T/F viruses (Figure 3F). Taken together these data confirm the correlation between virus entry and RT products detected by our assays and although T/F viruses show detectable entry defect into macrophages, this defect seems not to account for the overall low replication in macrophages.

### Reverse transcription is the primary block to clinical isolates

**Figure 4:**
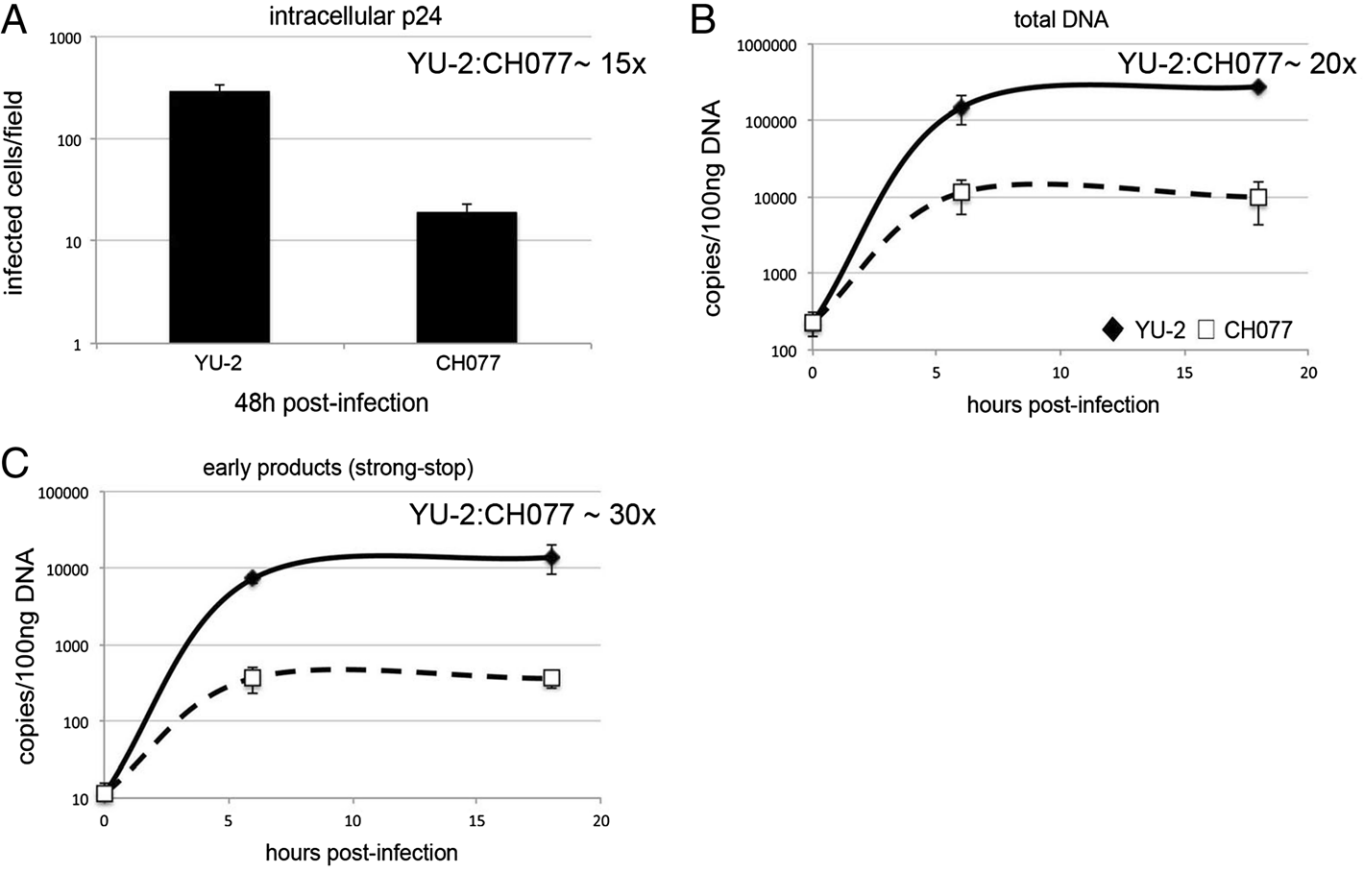
**Reverse transcription is additionally impaired in clinically derived transmitted/founder viruses.** MDM were from the same donor infected with equal amounts of p24 YU-2 and CH077 viruses. **(A)** MDM were fixed and stained for intracellular p24 protein 48 h post-infection. **(B,C)** MDM were harvested and total DNA was isolated at 0 h, 6 h and 18 h post-infection. Viral DNA products were detected using quantitative PCR. **(B)** total DNA; **(C)** strong stop. This is a representative example of three independent experiments on different donors. The YU-2:CH077 ratio represents fold difference in infection or viral DNA copies numbers between these viruses.

Having established that a modest entry defect appears to result in disproportionately compromised total DNA and p24 production, we next assessed how early in virus life cycle the block occurs. We infected MDM with a full-length clinical isolate, determined infection by intracellular p24 staining at 48 h post-infection (Figure 4A) and extracted total DNA at 0, 6 and 18 h post-infection for qPCR measurement of RT products (Figure 4B,C). Infection was reduced in the CH077 clinical isolate when compared to YU-2 (15 fold, Figure 4A), and accordingly there was a 20 fold difference in total HIV-1 DNA (Figure 4B). Interestingly, early viral DNA products (strong-stop, Figure 4C) also decreased by a similar magnitude, suggesting that the defect in RT is caused early in T/F viral life cycle. Moreover, this defect is evident early on (detected at 6 h) and did not change with time, suggesting that later steps of RT were not additionally impaired. We confirmed a consistent difference in early viral RT products (strong-stop) in multiple donors for two different T/F viruses (CH058, CH077) (Additional file 3: Figure S3). Together these data show that the difference in products of RT seems to be in concordance with the infection defect as assessed by intracellular p24 capsid protein (Figure 4A-C), and that clinically derived viruses are subject to post-entry restriction in macrophages relative to the macrophage tropic virus YU-2.

### The block to reverse transcription is not dissociable from entry using VSV-G

**Figure 5:**
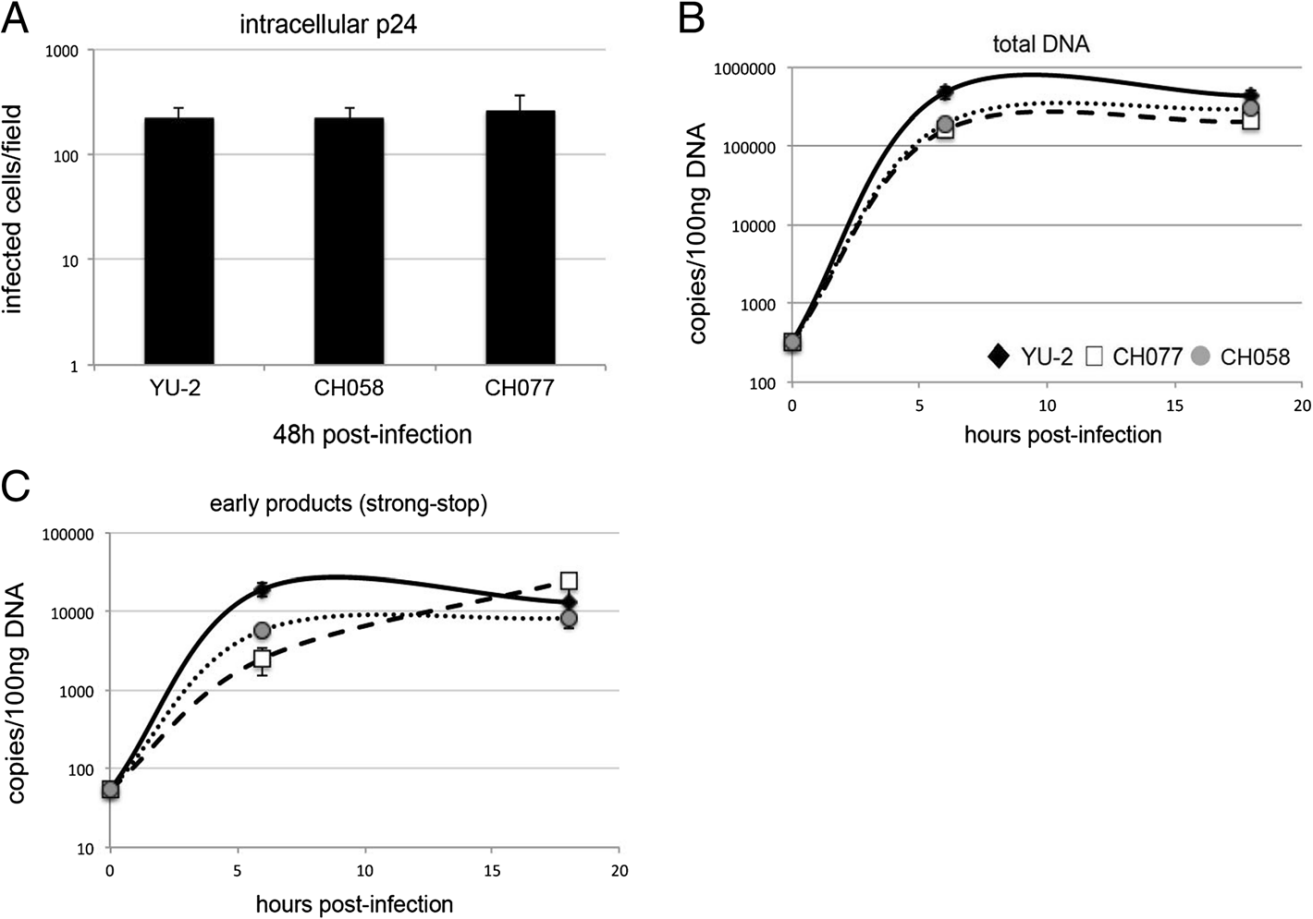
**Blocks to entry and reverse transcription are not dissociable from one another.** MDM from the same donor were infected with equal amounts of p24 VSV-G glycoprotein pseudotyped YU-2, CH058 and CH077 viruses. **(A)** MDM were fixed and stained for intracellular p24 protein 48 h post-infection. **(B,C)** MDM were harvested and total DNA was isolated at 0 h, 6 h and 18 h post-infection. Viral DNA products were detected using quantitative PCR. **(B)** total DNA; **(C)** strong stop. This is a representative example of three independent experiments on different donors.

In order to explore whether the entry and RT defects were dissociable from each other, we pseudotyped full-length YU-2, CH058 and CH077 with VSV-G glycoprotein and infected MDM with equal amounts of each virus. Intracellular p24 staining of MDM determined at 48 h post-infection (Figure 5A) showed equal overall infection for T/F viruses compared to YU-2. We detected a 4-8 fold difference in strong-stop and total viral DNA products at 6 h post-infection (p < 0.05), though this difference disappeared at 18 h post-infection (Figure 5B,C) (p = NS).

### Lack of evidence for tissue factors able to rescue RT

**Figure 6:**
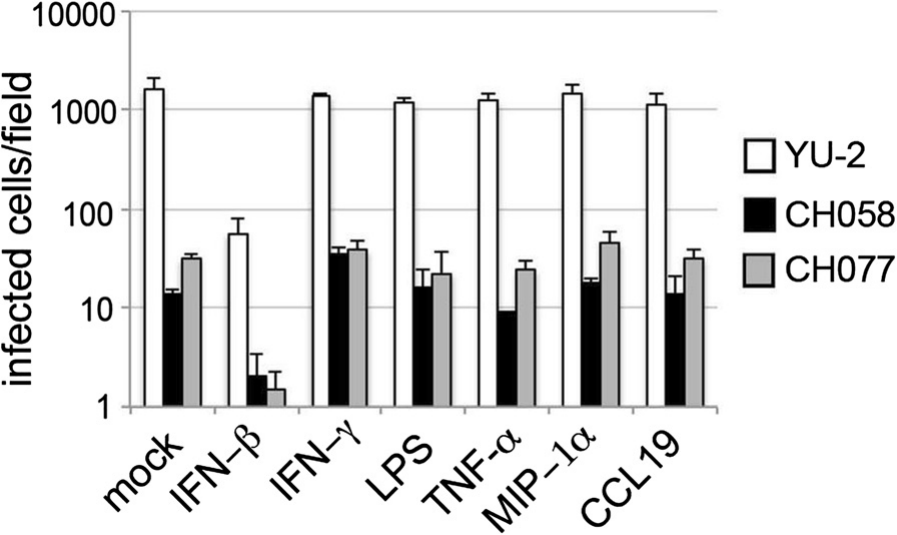
**Cytokines or LPS are not able to recapitulate the effect of Vpx in single round infection of MDM.** MDM were infected with equal amounts of p24 of YU-2 and subtype B full-length viruses CH077 and CH058 for 6 h. Cells were washed and new medium was added along with interferon beta (IFN-β; 5000U/ml), interferon gamma (IFN-γ; 20 ng/ml), LPS (100 ng/ml), tumor necrosis factor (TNF-α ; 10 ng/ml), MIP-1α (0.1 μg/ml), or CCL19 (100 ng/ml). This is a representative example of at least two independent experiments.

The tissue environment *in vivo* might contain factors which could mimic the effect of Vpx and relieve the post-entry block to clinically derived viruses. It would be important to know if post-entry viral cores within MDM could complete RT and infection, if the host macrophage were to enter a “conducive” environment such as lymphoid tissue. We tested this hypothesis by using a panel of stimuli encountered by macrophages *in vivo* during primary HIV infection [32], such as TNFα, interferon γ and β, ΜIP-1α and lipopolysaccharide (LPS). Furthermore we investigated the possible role of CCL19 (a CCR7 ligand), which has been suggested to play a role in promoting entry, RT and integration in unactivated T cells [33]. As our hypothesis was promotion of post-entry viral replication steps, we infected the cells with two clinically derived viruses and YU-2 for 6 h and after washing cells, used new medium supplemented with the indicated cytokines and LPS. Cells were examined for intracellular p24 staining 48 h post-infection. We did not observe significant changes in p24 production. The only exception was IFN-β, which caused significant decrease in infection of all three viruses tested, consistent with previous data [34] (Figure 6).

## Discussion

Macrophages are long-lived cells and have access to privileged anatomical sites. They are therefore potentially important cellular reservoirs. Macrophage tropic viruses are often present in CSF from patients with neurocognitive disease [4,35], but it is increasingly apparent that plasma derived viruses, even those able to use CCR5 receptors, replicate inefficiently in this cell type. We have sought to understand this apparent paradox by studying macrophage infection using viruses derived using SGA. All six full-length clinically derived transmitted/founder viruses studied showed low macrophage infection in single round infection compared to the macrophage tropic HIV-1 strain YU-2 or BaL, which have an intermediate to high tropism for macrophages [15].

Here we report for the first time that the sensitivity of clinically derived T/F viruses to Vpx complementation is similar to that described for laboratory strains [26,36]. Moreover, we showed that Vpx increased infection of all the T/F viruses in MDM by a similar magnitude as compared to YU-2. We observed differences in the level of infection in MDM after Vpx complementation between individual T/F viruses, although there was no statistically significant difference in Vpx mediated augmentation between subtype B and C viruses (p = 0.23). Surprisingly, rescue could be achieved even 24 h post-infection, arguing against the recently suggested nuclease activity for SAMHD1 acting on incoming viral RNA [37].

Although we have tested T/F viruses, envelopes from clinically derived chronic viruses show similar low levels of macrophage infection and therefore we believe our findings are also relevant to chronic infection [38]. Even though we were able to rescue extremely low infection in macrophages through Vpx complementation, there was still at least a 10 fold difference between infection by YU-2 and T/F clinical viruses in the presence of Vpx. Based on previous assumptions that low replication capacity in macrophages is due to a block in virus entry, these viruses should not have been rescued in our experiment.

Given that infection of T/F viruses in macrophages is not well defined, we proceeded to investigate this in a comprehensive way. We demonstrate that the block to infection in macrophages is sensitive to CD4 cell surface levels using Affinofile cells [31]. This cell assay system has not previously been used to examine the panel of full-length clinical isolates with Env expressed from the cognate provirus. Consistent with inefficient CD4 use, we confirmed there was a 2.5-4.5 fold reduction in viral entry to macrophages compared to YU-2 or BaL. This entry defect did not account for the whole replication defect of T/F viruses in MDM. We performed multiple controls showing that observed fold differences in MDM infection between T/F viruses and YU-2 or BaL cannot be related to problems with resolution of the entry assay relative to the RT qPCR assay, but rather represents an additional early post-entry block. This block seems to be at early RT or post-entry step. Importantly, this finding is in concordance with previous early reports: Potash et al. assessed fusion/entry in alveolar macrophages using a dequenching assay, revealing less than two fold difference between the AD8 macrophage tropic isolate and a ‘T-tropic isolate’, despite a much greater difference in cDNA and p24 production [39]. Also both the Desrosiers and Fauci laboratories concluded that the dominant restriction in human macrophages of SIVmac239 and the ‘T cell tropic’ virus 92 MW959 is determined by the viral envelope, but not at virus entry itself in macrophages [40,41]; post entry restriction was reported for the X4 viruses LAI and NDK in macrophages though entry was not assessed [42] and the predominant block was reported as being at nuclear translocation. Finally, the McKnight and Overbaugh groups have observed Env dependent post entry restriction in cell lines [43-45].

We demonstrate that the entry and RT blocks are not dissociable for currently circulating R5 using HIV strains, using VSV glycoprotein pseudotyped viral particles. These data suggest the post-entry restriction of T/F viruses in macrophages is largely dependent on the route of entry and interaction of viral envelope with CD4 receptor and co-receptors. VSV-G pseudotyping mediates viral entry through endocytosis, bypassing the requirement for the cell surface receptor CD4 and co-receptors CCR5 or CXCR4. Interestingly, HIV-1 envelope has been reported to induce intracellular signaling in CD4^+^ T cells to support viral replication [46,47]. Our observations are also highly reminiscent of data demonstrating that increases in surface CCR5 expression can lead to disproportionately large RT changes compared to entry changes [48], possibly related to signaling via calcium influx [41]. We speculate that inefficient CD4 and CCR5 usage by viruses (such as T/F viruses) may not only influence membrane fusion and entry, but also downstream events such as reverse transcription through intracellular signaling cascades. Interestingly, the actin cytoskeleton has been proposed as a barrier to RT [49], with depolymerizing agents able to increase infectivity and compensate for Nef deleted virus [50], as can VSV-G pseudotyping [51,52]. We are currently investigating the role of the actin cytoskeleton on T/F infection in macrophages.

Our study has important reservoir implications. Even though T/F viruses are inefficient in infecting MDM there is still low-level infection which, as we demonstrated, can be rescued by Vpx. It might be possible that subsequent changes in macrophage environment and state, for example activation due to concurrent infection or other inflammatory processes, might trigger changes that allow initiation of productive infection *in vivo*[53,54]. We therefore tested a panel of cytokines which might be encountered by macrophages *in vivo* during primary HIV infection [32]. Although none of these molecules showed any effect on clinically derived T/F viruses or YU-2 in a single round infection, there are likely to be tissue factors *in vivo* with similar post-entry effects as Vpx on HIV in macrophages.

There has been considerable controversy regarding the first targets of HIV infection following mucosal exposure, with CD4+ T cells and macrophages being the primary candidates. The realization that founder viruses were poorly macrophage tropic has resulted in a shift in the balance of opinion in favour of T cells. Our data showing that the virus core/reverse transcription complexes indeed enter the macrophage and are intact and biologically competent transform the discussion. In the transmission setting, occult infection of macrophages at the mucosa might occur without antigen presentation, with subsequent ‘reactivation’ in lymph nodes and efficient spread to other cell types.

## Conclusions

We propose that HIV-1 infected patients are likely at any given time to have a proportion of macrophages that contain HIV RNA/DNA species in the absence of productive infection. These macrophages may represent a reservoir and an important cellular target for HIV despite low levels of p24 *in vitro*. A greater understanding of macrophage infection using relevant viruses may be critical in curative strategies, which to date have focused largely on reactivation of latently infected T cells to target the HIV reservoir.

## Methods

### Reagents, antibodies, plasmids

Tissue culture media and supplements were obtained from Invitrogen (Paisley, UK), and tissue culture plastic was purchased from TPP (Trasadingen, Switzerland). All chemicals were purchased from Sigma (St. Louis, MO, USA) unless indicated otherwise. All infectious molecular clones were obtained from NIH AIDS Research and Reference Reagent Program (Germantown, MD, USA).

### Cell lines

293 T cells were cultured in DMEM complete (DMEM supplemented with 100 U/ml penicillin, 0.1 mg/ml streptomycin, and 10% FCS); 293-Affinofile cells were a kind gift from Benhur Lee and cultured in DMEM complete supplemented with 50 μg/ml of Blasticidin. CEM.NKR-CCR5-Luc [55] were cultured in RPMI complete (RPMI 1640, 100 U/ml penicillin, 0.1 mg/ml streptomycin, and 10% FCS) supplemented with 0.8 μg/ml of Gentamycin.

### Generation of virus stocks

Virus stocks were generated by DNA plasmid transfection of 293 T using Fugene HD (Promega UK Ltd, UK) according to the manufacture’s protocol. The VSV-G-pseudotyped virus was produced by co-transfection of 293 T with pMDG plasmid and YU-2, CH058 and CH077 HIV-1 molecular clones. Viral supernatant were harvest 48 h post-transfection, filtered through 0.45 μm pore-size filters and stored at -80°C. Clarified viral supernatant were analyzed by p24 ELISA (AIDS and Cancer Virus Program NCI-Frederick, MD, USA) for HIV-1 p24 antigen concentration and infectivity of virus stocks also compared using TZMBL indicator cells. Virus like particles containing Vpx were prepared as previously described [36].

### Monocyte isolation and differentiation

PBMC were prepared from HIV seronegative donors (after informed consent was obtained), by density-gradient centrifugation (Lymphoprep, Axis-Shield, UK). Monocyte-derived macrophages (MDM) were prepared by adherence with washing of non-adherent cells after 2 h, with subsequent maintenance of adherent cells in medium containing RPMI 1640 supplemented with 10% human AB serum (Sigma) and MCSF (10 ng/ml) for 3 days and then cultured for further 4 days in RPMI 1640 supplemented with 10% human AB serum.

### Infection of primary cells

1 × 10^5^ MDM were infected with 50 ng of p24 of each virus for 6 h at 37˚C. Cells were washed in PBS and new medium was added. MDM were fixed in ice cold acetone-methanol (1:1 [vol/vol]) 2 days post-infection, and infected cells identified by staining for p24 protein using a 1:1 mixture of the anti-p24 monoclonal antibodies EVA365 and EVA366 (NIBSC, Center for AIDS Reagents, UK) and a secondary goat anti-mouse beta-galactosidase-conjugated antibody (SouthernBiotech, AL, USA), and visualized by X-Gal (5-bromo-4-chloro-3-indolyl-β-d-galactopyranoside) staining (Promega). Infected cells were detected by light microscopy and counted as number of infected (blue) cells per inspected field.

### Induction and infection of 293-Affinofile cells

CD4 and CCR5 expression on the cell surface was induced as described previously [30]. Briefly, expression of CD4 was induced by doxycycline (0.1 ng/ml for low and 20 ng/ml for high CD4 expression) and CCR5 expression by ponasterone A (2 μM for high CCR5 expression) at 37˚C for 18 h. Cells were infected with 50 ng of p24 of each virus and spinoculated at 1200 g/2 h, room temperature. The infection medium was replaced with DMEM complete supplemented with 50 μg/ml of Blasticidin, and the cells were incubated for 48 h. Cells were washed, fixed in 3% paraformaldehyde, permeabilized with saponin and stained with anti HIV-1 p24 FITC-conjugated monoclonal antibody (Insight Biotechnology LTD, UK). Percentage of infection was determined by flow cytometry using BD FACSCalibur (BD Biosciences, UK) and analyzed by CellQuest (BD Biosciences) and FlowJo software (Tree Star, OR, USA).

### Measurement of HIV-1 entry (BlaM-Vpr assay)

MDM or CEM.NKR-CCR5-Luc cells were infected with 100 ng of HIV-1 virions containing BlaM-Vpr. Cells were spinoculated at 1200 g/2 h and further incubated for additional 2 h at 37˚C, 5%CO_2_, washed in CO_2_-independent medium and then loaded with CCF2-AM dye (Invitrogen) according to manufacture’s protocol in CO_2_-independent medium supplemented with 2.5 mM Probenicid (organic anion transport inhibitor). Cells were incubated for 1 h at room temperature, washed twice in CO_2_-independent medium and BlaM reaction was allowed to develop for 16 h in CO_2_-independent medium supplemented with 2.5 mM Probenicid at room temperature. Cells were washed in PBS, fixed in 3% paraformaldehyde and monitored by flow cytometry. Change in emission fluorescence of CCF2 (green to blue) after cleavage by the beta-lactamase (BlaM) was analyzed using BD LSRFortessa (BD Biosciences), FACSDIVA software (BD Biosciences) and FlowJo software (Tree Star).

### Quantitative PCR for early RT products

2 × 10^5^ MDM were infected with 100 ng of p24 of DNaseI-treated viruses for 0, 6, 18 h. Cell were washed and harvested for DNA isolation. Total DNA was extracted using the Qiagen DNeasy kit (Qiagen Ltd., UK) with the following modifications, cells were lysed in AL buffer and Proteinase K for 30 minutes at 56˚C and nucleic acids were eluted in 45 μl nuclease free molecular grade water (Promega) by heating the column at 37˚C for 10 minutes before centrifugation. 100 ng of DNA was analyzed by quantitative real time PCR for each of the following PCRs; total HIV DNA, strong stop DNA products. All reactions were performed in duplicate.

Primers and probe previously described [56] were used to detect strong stop products: oHC64 (TAACTAGGGAACCCACTGC) and oHC65 (GCTAGAGATTTTCCACACTG) and probe oHC66 (FAM-ACACAACAGACGGGCACACACTA-TAMRA). A final reaction contained Qiagen Quantitect Probe PCR master mix, 900nM of each primer and 250nM of probe. Cycling conditions were 95°C for 15 min, followed by 40 cycles of 15 s at 95°C and 1 min at 60°C. The reaction was performed on an Eppendorf Mastercycler ep realplex (Eppendorf UK Limited, UK). A dilution series of a YU-2 full-length molecular clone was used to create a standard curve.

### Total HIV DNA quantitation

Primers and probe were used to detect total DNA by amplifying the region between LTR and gag:HIV1LTR1(GCCTCAATAAAGCTTGCCTTGA),HIV1LTR2:(GGCGCCACTGCTAGAGATTTT) and HIV1LTRPR (FAM -TGTGACTCT GGTAACTAGAGATCCCTCAGAC-TAMRA). Additionally primers and probe for human pyruvate dehydrogenase (PDH) were duplexed in the reaction as an internal control. The primers and probe are as follows: PDH1 (TGAAAGTTATACAAAATTGAGGTCACTGTT),PDH2(TCCACAGCCCTCGACTAACC), PDHPR (JOE-CCCCCAGATACACTT AAGGGATCAACTCTTAATTGT-TAMRA). A final reaction contained Qiagen Multiplex PCR kit, 100nM each of the PDH primers and both the PDH and LTR probe as well as 200nM of LTR primers. Cycling conditions were 95°C for 15 min, followed by 45 cycles of 1 min at 94°C and 1 min at 60°C. The reaction was performed on an Eppendorf Mastercycler ep realplex. Extracted DNA from the 8E5 cell line, which contains one defective provirus per cell [57], was quantified and a dilution series was used to create a standard curve for both PDH and LTR-Gag.

### Statistical analysis

Student’s t-test was used to compare means. Statistical analyses were carried out in GraphPad Prism 5 (GraphPad Software Inc., La Jolla, California, USA).

## Competing interests

The authors declare that they have no competing interests.

## Authors’ contributions

RKG designed research, analyzed data, and wrote manusript; PM designed research, performed research, analyzed data and wrote manusript. SAW performed research and analysed data, MN designed research, analyzed data and provided reagents and protocols. GJT designed research and analyzed data. All authors read and approved the manuscript.

## Supplementary Material

Additional file 1: Figure S1 Similar infectivity across all viruses tested in primary T cells and the T cell line. Primary CD4+ T cells were isolated from PBMC using negative selection with antibody-coated magnetic beads (Miltenyi Biotec Ltd., UK) and stimulated for 3 days in the presence of PHA (at 2ug/ml) and IL-2 (at 10 pg/ml) in RPMI medium supplemented with 10% fetal calf serum. CD4+ T cells and CEM.NKR-CCR5-Luc (T cell line) expressing both CD4 and CCR5 receptors were infected with equal amounts of p24 of control virus YU-2 and subtype C and B full-length viruses for 6 hours. Cells were washed and new medium was added. (A) Supernatants from infected CD4+ T cells were collected and analyzed using p24 ELISA assay 4 days post-infection. (B) CEM.NKR-CCR5-Luc cells were lysed by adding Steady-Glo luciferase reagent (Promega, UK) 3 days post-infection and luminescence was read using a GloMax 96 Luminometer (Promega, UK). Data shown are mean of three independent experiments and error bars represent the standard deviation. Click here for file

Additional file 2: Figure S2 MDM infection by Env deficient virus. (A) MDM were infected with equal amounts of p24 of BlaM-Vpr containing viruses for 4 h. Cells were loaded with CCF2/AM dye and fusion events were detected by flow cytometry using BD LSR *Fortessa*, and gated from 10,000 cells. Percentage in each panel represents virus fusion positive cells (cleaved CCF2). (B) MDM were infected with 50 ng of p24 of virus for 4 h. Cells were washed in PBS and new medium was added. MDM were fixed in ice cold acetone-methanol (1:1 [vol/vol]) 48 h post-infection, and infected cells identified by staining for intracellular p24 protein. Un-infected: un-infected control; ZM247Fv1Δenv: envelope deficient virus; ZM247Fv1Δenv + YU-2: envelope deficient virus complemented with YU-2 envelope. Data shown are representative example of two independent experiments. Click here for file

Additional file 3: Figure S3 Early reverse transcription efficiency. MDM from three different donors were infected with equal amount of p24 YU-2, CH058 and CH077 for 6 h. Cells were harvested and total DNA was isolated. Early viral DNA products were detected (strong stop) using quantitative PCR (see in methods: Quantitative PCR for early RT products). All the experiments were conducted in duplicate. Click here for file

## References

[B1] Koppensteiner H Brack-Werner R Schindler M Macrophages and their relevance in human immunodeficiency virus type I infection Retrovirology 2012 9 82 10.1186/1742-4690-9-82 23035819 PMC3484033

[B2] Simioni S Cavassini M Annoni JM Rimbault Abraham A Bourquin I Schiffer V Calmy A Chave JP Giacobini E Hirschel B Du Pasquier RA Cognitive dysfunction in HIV patients despite long-standing suppression of viremia AIDS 2010 24 9 1243 1250 19996937 10.1097/QAD.0b013e3283354a7b

[B3] Antinori A Perno CF Giancola ML Forbici F Ippolito G Hoetelmans RM Piscitelli SC Efficacy of cerebrospinal fluid (CSF)-penetrating antiretroviral drugs against HIV in the neurological compartment: different patterns of phenotypic resistance in CSF and plasma Clin Infect Dis 2005 41 12 1787 1793 10.1086/498310 16288405

[B4] Schnell G Joseph S Spudich S Price RW Swanstrom R HIV-1 replication in the central nervous system occurs in two distinct cell types PLoS Pathog 2011 7 10 e1002286 10.1371/journal.ppat.1002286 22007152 PMC3188520

[B5] Schnell G Spudich S Harrington P Price RW Swanstrom R Compartmentalized human immunodeficiency virus type 1 originates from long-lived cells in some subjects with HIV-1-associated dementia PLoS Pathog 2009 5 4 e1000395 10.1371/journal.ppat.1000395 19390619 PMC2668697

[B6] Reynoso R Wieser M Ojeda D Bönisch M Kühnel H Bolcic F Quendler H Grillari J Grillari-Voglauer R Quarleri J HIV-1 induces telomerase activity in monocyte-derived macrophages-safeguarding one of its reservoirs? J Virol 2012 86 19 10327 10337 10.1128/JVI.01495-12 22787205 PMC3457250

[B7] Gray L Sterjovski J Churchill M Ellery P Nasr N Lewin SR Crowe SM Wesselingh SL Cunningham AL Gorry PR Uncoupling coreceptor usage of human immunodeficiency virus type 1 (HIV-1) from macrophage tropism reveals biological properties of CCR5-restricted HIV-1 isolates from patients with acquired immunodeficiency syndrome Virology 2005 337 2 384 398 10.1016/j.virol.2005.04.034 15916792

[B8] Goodenow MM Collman RG HIV-1 coreceptor preference is distinct from target cell tropism: a dual-parameter nomenclature to define viral phenotypes J Leukoc Biol 2006 80 5 965 972 10.1189/jlb.0306148 16923919

[B9] Peters PJ Sullivan WM Duenas-Decamp MJ Bhattacharya J Ankghuambom C Brown R Luzuriaga K Bell J Simmonds P Ball J Clapham PR Non-macrophage-tropic human immunodeficiency virus type 1 R5 envelopes predominate in blood, lymph nodes, and semen: implications for transmission and pathogenesis J Virol 2006 80 13 6324 6332 10.1128/JVI.02328-05 16775320 PMC1488974

[B10] Peters PJ Bhattacharya J Hibbitts S Dittmar MT Simmons G Bell J Simmonds P Clapham PR Biological analysis of human immunodeficiency virus type 1 R5 envelopes amplified from brain and lymph node tissues of AIDS patients with neuropathology reveals two distinct tropism phenotypes and identifies envelopes in the brain that confer an enhanced tropism and fusigenicity for macrophages J Virol 2004 78 13 6915 6926 10.1128/JVI.78.13.6915-6926.2004 15194768 PMC421670

[B11] Musich T Musich T Peters PJ Duenas-Decamp MJ Gonzalez-Perez MP Robinson J Zolla-Pazner S Ball JK Luzuriaga K Clapham PR A conserved determinant in the V1 loop of HIV-1 modulates the V3 loop to prime low CD4 use and macrophage infection J Virol 2011 85 5 2397 2405 10.1128/JVI.02187-10 21159865 PMC3067776

[B12] Dunfee RL Thomas ER Gorry PR Wang J Taylor J Kunstman K Wolinsky SM Gabuzda D The HIV Env variant N283 enhances macrophage tropism and is associated with brain infection and dementia Proc Natl Acad Sci U S A 2006 103 41 15160 15165 10.1073/pnas.0605513103 17015824 PMC1586182

[B13] Duenas-Decamp MJ Peters PJ Burton D Clapham PR Determinants flanking the CD4 binding loop modulate macrophage tropism of human immunodeficiency virus type 1 R5 envelopes J Virol 2009 83 6 2575 2583 10.1128/JVI.02133-08 19129457 PMC2648272

[B14] Keele BF Identifying and characterizing recently transmitted viruses Curr Opin HIV AIDS 2010 5 4 327 334 10.1097/COH.0b013e32833a0b9b 20543609 PMC2914479

[B15] Salazar-Gonzalez JF Salazar MG Keele BF Learn GH Giorgi EE Li H Decker JM Wang S Baalwa J Kraus MH Parrish NF Shaw KS Guffey MB Bar KJ Davis KL Ochsenbauer-Jambor C Kappes JC Saag MS Cohen MS Mulenga J Derdeyn CA Allen S Hunter E Markowitz M Hraber P Perelson AS Bhattacharya T Haynes BF Korber BT Hahn BH Shaw GM Genetic identity, biological phenotype, and evolutionary pathways of transmitted/founder viruses in acute and early HIV-1 infection J Exp Med 2009 206 6 1273 1289 10.1084/jem.20090378 19487424 PMC2715054

[B16] Haaland RE Hawkins PA Salazar-Gonzalez J Johnson A Tichacek A Karita E Manigart O Mulenga J Keele BF Shaw GM Hahn BH Allen SA Derdeyn CA Hunter E Inflammatory genital infections mitigate a severe genetic bottleneck in heterosexual transmission of subtype A and C HIV-1 PLoS Pathog 2009 5 1 e1000274 10.1371/journal.ppat.1000274 19165325 PMC2621345

[B17] Vermeire J Vanbillemont G Witkowski W Verhasselt B The Nef-infectivity enigma: mechanisms of enhanced lentiviral infection Curr HIV Res 2011 9 7 474 489 10.2174/157016211798842099 22103831 PMC3355465

[B18] Lopez-Verges S Camus G Blot G Beauvoir R Benarous R Berlioz-Torrent C Tail-interacting protein TIP47 is a connector between Gag and Env and is required for Env incorporation into HIV-1 virions Proc Natl Acad Sci U S A 2006 103 40 14947 14952 10.1073/pnas.0602941103 17003132 PMC1595456

[B19] Bhatia AK Kaushik R Campbell NA Pontow SE Ratner L Mutation of critical serine residues in HIV-1 matrix result in an envelope incorporation defect which can be rescued by truncation of the gp41 cytoplasmic tail Virology 2009 384 1 233 241 10.1016/j.virol.2008.10.047 19059618 PMC2651518

[B20] Li M Salazar-Gonzalez JF Derdeyn CA Morris L Williamson C Robinson JE Decker JM Li Y Salazar MG Polonis VR Mlisana K Karim SA Hong K Greene KM Bilska M Zhou J Allen S Chomba E Mulenga J Vwalika C Gao F Zhang M Korber BT Hunter E Hahn BH Montefiori DC Genetic and neutralization properties of subtype C human immunodeficiency virus type 1 molecular env clones from acute and early heterosexually acquired infections in Southern Africa J Virol 2006 80 23 11776 11790 10.1128/JVI.01730-06 16971434 PMC1642599

[B21] Parrish NF Wilen CB Banks LB Iyer SS Pfaff JM Salazar-Gonzalez JF Salazar MG Decker JM Parrish EH Berg A Hopper J Hora B Kumar A Mahlokozera T Yuan S Coleman C Vermeulen M Ding H Ochsenbauer C Tilton JC Permar SR Kappes JC Betts MR Busch MP Gao F Montefiori D Haynes BF Shaw GM Hahn BH Doms RW Transmitted/founder and chronic subtype C HIV-1 use CD4 and CCR5 receptors with equal efficiency and are not inhibited by blocking the integrin alpha4beta7 PLoS Pathog 2012 8 5 e1002686 10.1371/journal.ppat.1002686 22693444 PMC3364951

[B22] Wilen CB Parrish NF Pfaff JM Decker JM Henning EA Haim H Petersen JE Wojcechowskyj JA Sodroski J Haynes BF Montefiori DC Tilton JC Shaw GM Hahn BH Doms RW Phenotypic and immunologic comparison of clade B transmitted/founder and chronic HIV-1 envelope glycoproteins J Virol 2011 85 17 8514 8527 10.1128/JVI.00736-11 21715507 PMC3165820

[B23] Ochsenbauer C Edmonds TG Ding H Keele BF Decker J Salazar MG Salazar-Gonzalez JF Shattock R Haynes BF Shaw GM Hahn BH Kappes JC Generation of transmitted/founder HIV-1 infectious molecular clones and characterization of their replication capacity in CD4 T lymphocytes and monocyte-derived macrophages J Virol 2012 86 5 2715 2728 10.1128/JVI.06157-11 22190722 PMC3302286

[B24] Laguette N Sobhian B Casartelli N Ringeard M Chable-Bessia C Ségéral E Yatim A Emiliani S Schwartz O Benkirane M SAMHD1 is the dendritic- and myeloid-cell-specific HIV-1 restriction factor counteracted by Vpx Nature 2011 474 7353 654 657 10.1038/nature10117 21613998 PMC3595993

[B25] Hrecka K Hao C Gierszewska M Swanson SK Kesik-Brodacka M Srivastava S Florens L Washburn MP Skowronski J Vpx relieves inhibition of HIV-1 infection of macrophages mediated by the SAMHD1 protein Nature 2011 474 7353 658 661 10.1038/nature10195 21720370 PMC3179858

[B26] Lahouassa H Daddacha W Hofmann H Ayinde D Logue EC Dragin L Bloch N Maudet C Bertrand M Gramberg T Pancino G Priet S Canard B Laguette N Benkirane M Transy C Landau NR Kim B Margottin-Goguet F SAMHD1 restricts the replication of human immunodeficiency virus type 1 by depleting the intracellular pool of deoxynucleoside triphosphates Nat Immunol 2012 13 3 223 228 10.1038/ni.2236 22327569 PMC3771401

[B27] Fiebig EW Wright DJ Rawal BD Garrett PE Schumacher RT Peddada L Heldebrant C Smith R Conrad A Kleinman SH Busch MP Dynamics of HIV viremia and antibody seroconversion in plasma donors: implications for diagnosis and staging of primary HIV infection Aids 2003 17 13 1871 1879 10.1097/00002030-200309050-00005 12960819

[B28] Lee B Sharron M Montaner LJ Weissman D Doms RW Quantification of CD4, CCR5, and CXCR4 levels on lymphocyte subsets, dendritic cells, and differentially conditioned monocyte-derived macrophages Proc Natl Acad Sci U S A 1999 96 9 5215 5220 10.1073/pnas.96.9.5215 10220446 PMC21844

[B29] Duncan CJ Sattentau QJ Viral determinants of HIV-1 macrophage tropism Viruses 2011 3 11 2255 2279 22163344 10.3390/v3112255 PMC3230851

[B30] Johnston SH Lobritz MA Nguyen S Lassen K Delair S Posta F Bryson YJ Arts EJ Chou T Lee B A quantitative affinity-profiling system that reveals distinct CD4/CCR5 usage patterns among human immunodeficiency virus type 1 and simian immunodeficiency virus strains J Virol 2009 83 21 11016 11026 10.1128/JVI.01242-09 19692480 PMC2772777

[B31] Cavrois M De Noronha C Greene WC A sensitive and specific enzyme-based assay detecting HIV-1 virion fusion in primary T lymphocytes Nat Biotechnol 2002 20 11 1151 1154 10.1038/nbt745 12355096

[B32] Stacey AR Norris PJ Qin L Haygreen EA Taylor E Heitman J Lebedeva M DeCamp A Li D Grove D Self SG Borrow P Induction of a striking systemic cytokine cascade prior to peak viremia in acute human immunodeficiency virus type 1 infection, in contrast to more modest and delayed responses in acute hepatitis B and C virus infections J Virol 2009 83 8 3719 3733 10.1128/JVI.01844-08 19176632 PMC2663284

[B33] Saleh S Solomon A Wightman F Xhilaga M Cameron PU Lewin SR CCR7 ligands CCL19 and CCL21 increase permissiveness of resting memory CD4+ T cells to HIV-1 infection: a novel model of HIV-1 latency Blood 2007 110 13 4161 4164 10.1182/blood-2007-06-097907 17881634

[B34] Tsang J Tsang J Chain BM Miller RF Webb BL Barclay W Towers GJ Katz DR Noursadeghi M HIV-1 infection of macrophages is dependent on evasion of innate immune cellular activation Aids 2009 23 17 2255 2263 10.1097/QAD.0b013e328331a4ce 19741482 PMC2873676

[B35] Sturdevant CB Dow A Jabara CB Joseph SB Schnell G Takamune N Mallewa M Heyderman RS Van Rie A Swanstrom R Central nervous system compartmentalization of HIV-1 subtype C variants early and late in infection in young children PLoS Pathog 2012 8 12 e1003094 10.1371/journal.ppat.1003094 23300446 PMC3531524

[B36] Goujon C Arfi V Pertel T Luban J Lienard J Rigal D Darlix JL Cimarelli A Characterization of simian immunodeficiency virus SIVSM/human immunodeficiency virus type 2 Vpx function in human myeloid cells J Virol 2008 82 24 12335 12345 10.1128/JVI.01181-08 18829761 PMC2593360

[B37] Beloglazova N Flick R Tchigvintsev A Brown G Popovic A Nocek B Yakunin AF Nuclease activity of the human SAMHD1 protein implicated in the Aicardi-Goutieres syndrome and HIV-1 restriction J Biol Chem 2013 288 12 8101 8110 10.1074/jbc.M112.431148 23364794 PMC3605629

[B38] Ping LH Joseph SB Anderson JA Abrahams MR Salazar-Gonzalez JF Kincer LP Treurnicht FK Arney L Ojeda S Zhang M Keys J Potter EL Chu H Moore P Salazar MG Iyer S Jabara C Kirchherr J Mapanje C Ngandu N Seoighe C Hoffman I Gao F Tang Y Labranche C Lee B Saville A Vermeulen M Fiscus S Morris L Karim SA Haynes BF Shaw GM Korber BT Hahn BH Cohen MS Montefiori D Williamson C Swanstrom R CAPRISA Acute Infection Study and the Center for HIV-AIDS Vaccine Immunology Consortium Comparison of viral Env proteins from acute and chronic infections with subtype C human immunodeficiency virus type 1 identifies differences in glycosylation and CCR5 utilization and suggests a new strategy for immunogen design J Virol 2013 87 13 7218 7233 10.1128/JVI.03577-12 23616655 PMC3700278

[B39] Potash MJ Zeira M Huang ZB Pearce TE Eden E Gendelman HE Volsky DJ Virus-cell membrane fusion does not predict efficient infection of alveolar macrophages by human immunodeficiency virus type 1 (HIV-1) Virology 1992 188 2 864 868 10.1016/0042-6822(92)90543-X 1585653

[B40] Mori K Ringler DJ Desrosiers RC Restricted replication of simian immunodeficiency virus strain 239 in macrophages is determined by env but is not due to restricted entry J Virol 1993 67 5 2807 2814 7682627 10.1128/jvi.67.5.2807-2814.1993 PMC237605

[B41] Arthos J Rubbert A Rabin RL Cicala C Machado E Wildt K Hanbach M Steenbeke TD Swofford R Farber JM Fauci AS CCR5 signal transduction in macrophages by human immunodeficiency virus and simian immunodeficiency virus envelopes J Virol 2000 74 14 6418 6424 10.1128/JVI.74.14.6418-6424.2000 10864653 PMC112149

[B42] Schmidtmayerova H Alfano M Nuovo G Bukrinsky M Human immunodeficiency virus type 1 T-lymphotropic strains enter macrophages via a CD4- and CXCR4-mediated pathway: replication is restricted at a postentry level J Virol 1998 72 6 4633 4642 9573226 10.1128/jvi.72.6.4633-4642.1998 PMC109980

[B43] Schmitz C Marchant D Neil SJ Aubin K Reuter S Dittmar MT McKnight A Lv2, a novel postentry restriction, is mediated by both capsid and envelope J Virol 2004 78 4 2006 2016 10.1128/JVI.78.4.2006-2016.2004 14747565 PMC369432

[B44] Marchant D Neil SJ Aubin K Schmitz C McKnight A An envelope-determined, pH-independent endocytic route of viral entry determines the susceptibility of human immunodeficiency virus type 1 (HIV-1) and HIV-2 to Lv2 restriction J Virol 2005 79 15 9410 9418 10.1128/JVI.79.15.9410-9418.2005 16014904 PMC1181606

[B45] Pineda MJ Orton BR Overbaugh J A TRIM5alpha-independent post-entry restriction to HIV-1 infection of macaque cells that is dependent on the path of entry Virology 2007 363 2 310 318 10.1016/j.virol.2007.02.002 17350067 PMC2743720

[B46] Cicala C Cicala C Arthos J Selig SM Dennis G Jr Hosack DA Van Ryk D Spangler ML Steenbeke TD Khazanie P Gupta N Yang J Daucher M Lempicki RA Fauci AS HIV envelope induces a cascade of cell signals in non-proliferating target cells that favor virus replication Proc Natl Acad Sci U S A 2002 99 14 9380 9385 10.1073/pnas.142287999 12089333 PMC123149

[B47] Kinter AL Umscheid CA Arthos J Cicala C Lin Y Jackson R Donoghue E Ehler L Adelsberger J Rabin RL Fauci AS HIV envelope induces virus expression from resting CD4+ T cells isolated from HIV-infected individuals in the absence of markers of cellular activation or apoptosis J Immunol 2003 170 5 2449 2455 12594269 10.4049/jimmunol.170.5.2449

[B48] Lin YL Mettling C Portales P Reynes J Clot J Corbeau P Cell surface CCR5 density determines the postentry efficiency of R5 HIV-1 infection Proc Natl Acad Sci U S A 2002 99 24 15590 15595 10.1073/pnas.242134499 12434015 PMC137761

[B49] Bukrinskaya A Brichacek B Mann A Stevenson M Establishment of a functional human immunodeficiency virus type 1 (HIV-1) reverse transcription complex involves the cytoskeleton J Exp Med 1998 188 11 2113 2125 10.1084/jem.188.11.2113 9841925 PMC2212381

[B50] Campbell EM Nunez R Hope TJ Disruption of the actin cytoskeleton can complement the ability of Nef to enhance human immunodeficiency virus type 1 infectivity J Virol 2004 78 11 5745 5755 10.1128/JVI.78.11.5745-5755.2004 15140972 PMC415815

[B51] Aiken C Pseudotyping human immunodeficiency virus type 1 (HIV-1) by the glycoprotein of vesicular stomatitis virus targets HIV-1 entry to an endocytic pathway and suppresses both the requirement for Nef and the sensitivity to cyclosporin A J Virol 1997 71 8 5871 5877 9223476 10.1128/jvi.71.8.5871-5877.1997 PMC191842

[B52] Chazal N Singer G Aiken C Hammarskjöld ML Rekosh D Human immunodeficiency virus type 1 particles pseudotyped with envelope proteins that fuse at low pH no longer require Nef for optimal infectivity J Virol 2001 75 8 4014 4018 10.1128/JVI.75.8.4014-4018.2001 11264394 PMC114896

[B53] Lawn SD Roberts BD Griffin GE Folks TM Butera ST Cellular compartments of human immunodeficiency virus type 1 replication in vivo: determination by presence of virion-associated host proteins and impact of opportunistic infection J Virol 2000 74 1 139 145 10.1128/JVI.74.1.139-145.2000 10590100 PMC111522

[B54] Herbein G Varin A The macrophage in HIV-1 infection: from activation to deactivation? Retrovirology 2010 7 33 10.1186/1742-4690-7-33 20380696 PMC2859752

[B55] Spenlehauer C Gordon CA Trkola A Moore JP A luciferase-reporter gene-expressing T-cell line facilitates neutralization and drug-sensitivity assays that use either R5 or X4 strains of human immunodeficiency virus type 1 Virology 2001 280 2 292 300 10.1006/viro.2000.0780 11162843

[B56] Bishop KN Holmes RK Malim MH Antiviral potency of APOBEC proteins does not correlate with cytidine deamination J Virol 2006 80 17 8450 8458 10.1128/JVI.00839-06 16912295 PMC1563846

[B57] Clavel F Hoggan MD Willey RL Strebel K Martin MA Repaske R Genetic recombination of human immunodeficiency virus J Virol 1989 63 3 1455 1459 2915387 10.1128/jvi.63.3.1455-1459.1989 PMC247851

