# Supplementary figures and images for: Vpx complementation of ‘non-macrophage tropic’ R5 viruses reveals robust entry of infectious HIV-1 cores into macrophages
Source: Retrovirology. 2014 Mar 21;11:25. doi: 10.1186/1742-4690-11-25 (PMC3997928; doi:10.1186/1742-4690-11-25)

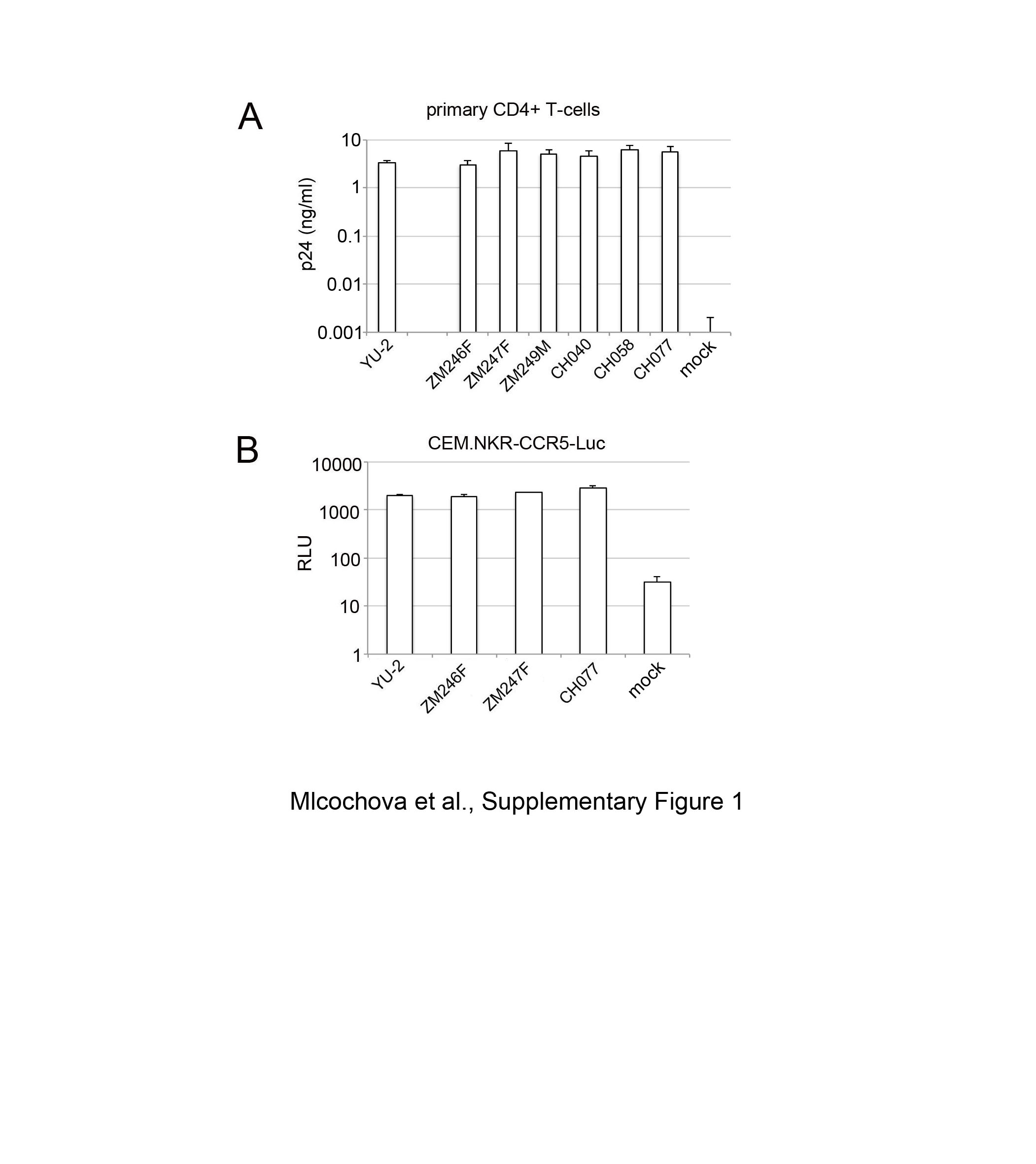

Supplement: Additional file 1: Figure S1 — Similar infectivity across all viruses tested in primary T cells and the T cell line. Primary CD4+ T cells were isolated from PBMC using negative selection with antibody-coated magnetic beads (Miltenyi Biotec Ltd., UK) and stimulated for 3 days in the presence of PHA (at 2ug/ml) and IL-2 (at 10 pg/ml) in RPMI medium supplemented with 10% fetal calf serum. CD4+ T cells and CEM.NKR-CCR5-Luc (T cell line) expressing both CD4 and CCR5 receptors were infected with equal amounts of p24 of control virus YU-2 and subtype C and B full-length viruses for 6 hours. Cells were washed and new medium was added. (A) Supernatants from infected CD4+ T cells were collected and analyzed using p24 ELISA assay 4 days post-infection. (B) CEM.NKR-CCR5-Luc cells were lysed by adding Steady-Glo luciferase reagent (Promega, UK) 3 days post-infection and luminescence was read using a GloMax 96 Luminometer (Promega, UK). Data shown are mean of three independent experiments and error bars represent the standard deviation. [file 1742-4690-11-25-S1.jpeg]

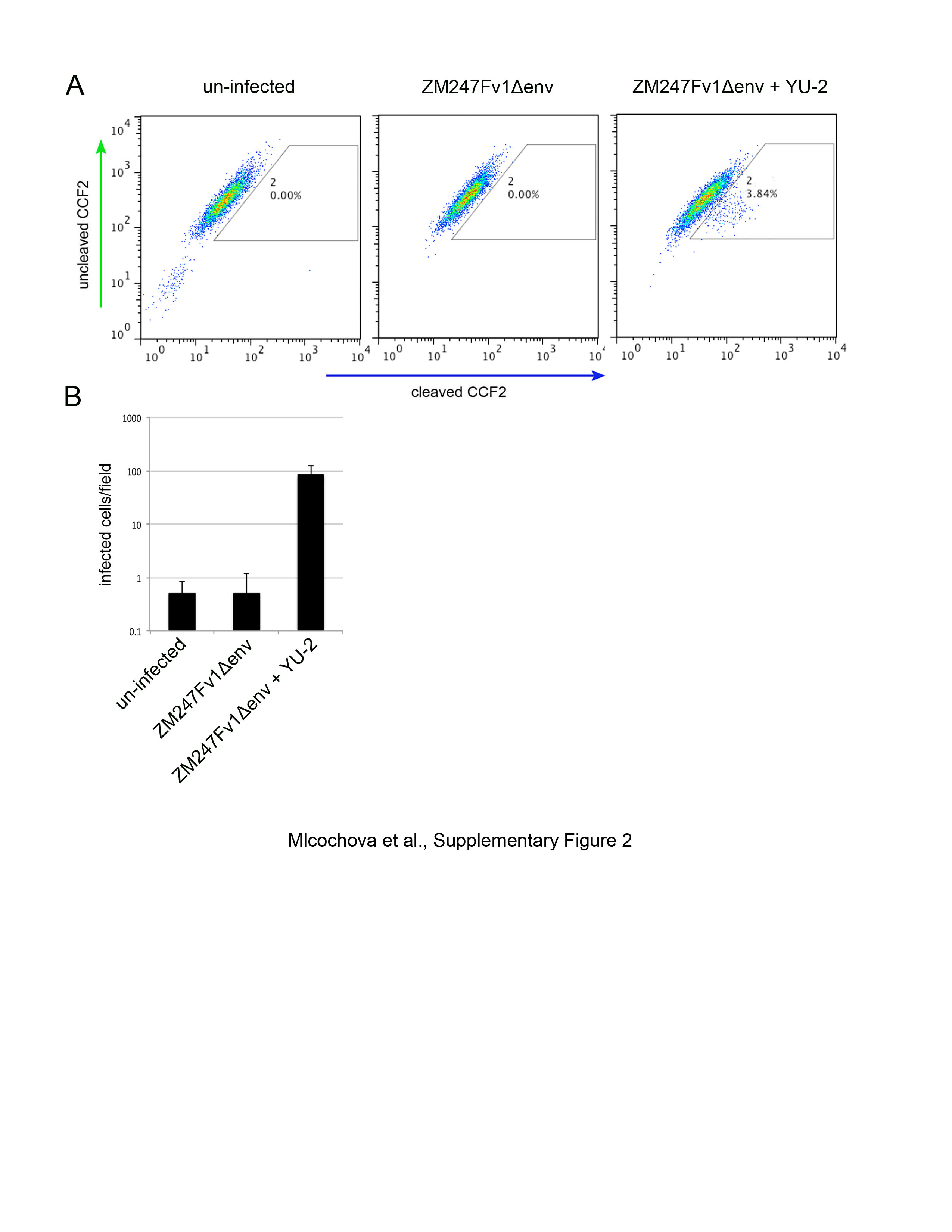

Supplement: Additional file 2: Figure S2 — MDM infection by Env deficient virus. (A) MDM were infected with equal amounts of p24 of BlaM-Vpr containing viruses for 4 h. Cells were loaded with CCF2/AM dye and fusion events were detected by flow cytometry using BD LSR Fortessa, and gated from 10,000 cells. Percentage in each panel represents virus fusion positive cells (cleaved CCF2). (B) MDM were infected with 50 ng of p24 of virus for 4 h. Cells were washed in PBS and new medium was added. MDM were fixed in ice cold acetone-methanol (1:1 [vol/vol]) 48 h post-infection, and infected cells identified by staining for intracellular p24 protein. Un-infected: un-infected control; ZM247Fv1Δenv: envelope deficient virus; ZM247Fv1Δenv + YU-2: envelope deficient virus complemented with YU-2 envelope. Data shown are representative example of two independent experiments. [file 1742-4690-11-25-S2.jpeg]

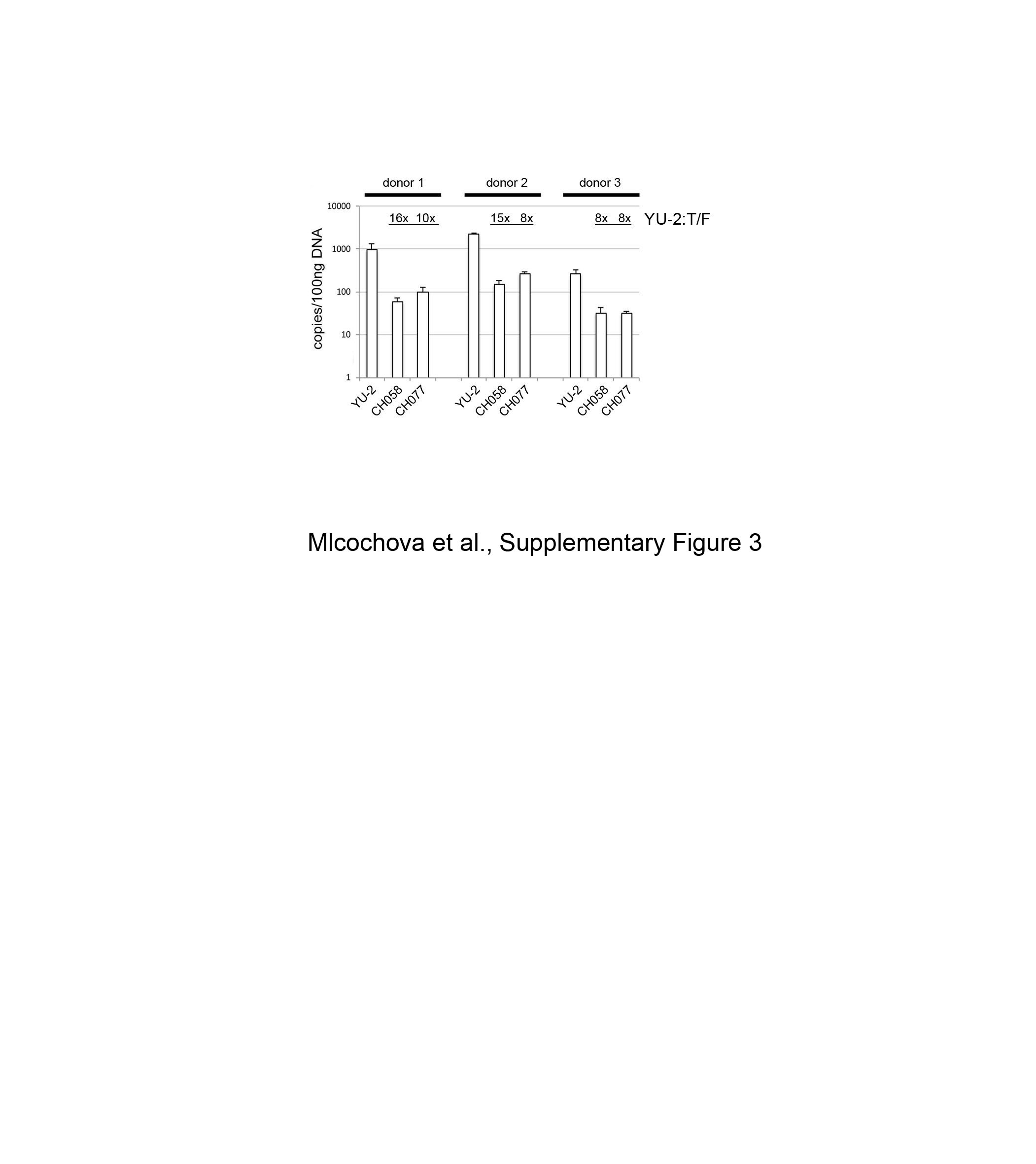

Supplement: Additional file 3: Figure S3 — Early reverse transcription efficiency. MDM from three different donors were infected with equal amount of p24 YU-2, CH058 and CH077 for 6 h. Cells were harvested and total DNA was isolated. Early viral DNA products were detected (strong stop) using quantitative PCR (see in methods: Quantitative PCR for early RT products). All the experiments were conducted in duplicate. [file 1742-4690-11-25-S3.jpeg]
